# Evaluation of inflammatory and cardiovascular-related indices as potential markers for postmenopausal osteoporosis—a retrospective study

**DOI:** 10.7717/peerj.21311

**Published:** 2026-06-24

**Authors:** Alihan Tigli, Yakup Baykus, Nazli Sener, Yasemin Ercan De­girmenci, Guzide Ece Akinci, Erdem Gurkan, Ca­gdas Colluog­lu, Rulin Deniz

**Affiliations:** 1 Faculty of Medicine, Department of Obstetrics and Gynaecology, Bandırma Onyedi Eylül University, Bandırma, Balıkesir, Turkey; 2 Gynecology and Obstetrics Clinic, Bandırma Training and Research Hospital, Bandırma, Balıkesir, Turkey; 3 Faculty of Medicine, Department of Obstetrics and Gynaecology, Başkent University, İzmir, Turkey

**Keywords:** Postmenopausal osteoporosis, Inflammatory markers, Biochemical indicators, Calcium, Menopause, Bone, Bone mineral density

## Abstract

**Background:**

Emerging evidence suggests a strong pathophysiological link between osteoporosis (OP), characterized by low bone mineral density (BMD), and cardiovascular disease. Biomarkers such as the atherogenic index of plasma (AIP) and the triglyceride-glucose (TyG) index, while primarily linked to cardiovascular risk, may also reflect the metabolic dysregulation seen in OP. Similarly, inflammatory markers including neutrophil-lymphocyte ratio (NLR), platelet-lymphocyte ratio (PLR), monocyte-lymphocyte ratio (MLR), and neutrophil-monocyte ratio (NMR), along with ratios like lymphocyte/HDL-C, monocyte/HDL-C, and granulocyte/HDL-C have been suggested as potential diagnostic tools. The aim of this study was to evaluate the diagnostic discrimination of these accessible biomarkers for postmenopausal OP.

**Methods:**

A retrospective analysis was performed on 387 postmenopausal women who underwent bone densitometry between 2021 and 2025. Data on body mass index (BMI), T-scores, age, menopause duration, and relevant laboratory parameters were collected. Inflammatory and biochemical indices were calculated using established formulas.

**Results:**

Age and menopause duration were significantly higher in the OP group compared to the normal and osteopenia groups (*p* < 0.01). Conversely, the median femoral neck T-score (FN-T) was significantly lower in the OP group. Among the biomarkers, only the monocyte/HDL-C ratio was significantly lower in the OP group (*p* < 0.05). Receiver operating characteristic (ROC) analysis indicated poor diagnostic discrimination for the monocyte/HDL-C ratio (AUC: 0.608), while all other biomarkers were non-significant (AUC < 0.6).

**Conclusion:**

Even the statistically significant monocyte/HDL-C ratio failed to achieve clinically acceptable discriminatory ability. Consequently, these biomarkers cannot be recommended as standalone screening tools for postmenopausal osteoporosis.

## Introduction

The onset of menopause is characterised by a decline in oestrogen production by the ovaries, which renders women susceptible to osteoporosis (OP), cardiovascular disease (CVD), and metabolic disorders (Camacho et al., 2016). OP is defined as a systemic disorder characterised by low bone mass and deterioration in the microarchitecture of bone tissue. Low bone mineral density (BMD) is a defining feature of OP (Rosano et al., 2007). Approximately 200 million people worldwide are affected by OP, which leads to an increase in bone fragility and causes nine million fractures annually (Baldini et al., 2005).

A robust correlation has been identified between OP and CVD in women afflicted with OP (Tankó et al., 2005). It is noteworthy that both diseases exhibit shared pathophysiological mechanisms, a phenomenon often termed the “common ground” hypothesis. The development of both diseases is significantly influenced by chronic low-grade inflammation and lipid metabolism disorders (Zhang et al., 2024). Proinflammatory cytokines, such as TNF-α and IL-6, have been shown to increase bone resorption by stimulating osteoclast activity, while also promoting arterial plaque formation (Szekanecz et al., 2019). Dyslipidaemia has been identified as a risk factor for cardiovascular disease. In addition, oxidised lipids have been observed to accumulate in bone tissue, thereby inhibiting osteoblast differentiation and reducing bone formation (Zhang et al., 2024). Decreased oestrogen levels, which are associated with menopause, have also been demonstrated to adversely affect lipid metabolism, impacting both bone health and the risk of CVD (Mou et al., 2025). In view of the aforementioned pathophysiological mechanisms, the investigation of biomarkers reflecting both inflammation and lipid metabolism may provide valuable insights into bone health.

In recent years, composite metabolic-inflammatory indices have gained importance as a reflection of these common mechanisms. The atherogenic plasma index (AIP), calculated as log(triglycerides/high-density lipoprotein (HDL) cholesterol), has been shown to be a marker that shows an inverse correlation with small, dense LDL particles. AIP has been shown to reflect the balance between atherogenic and protective lipoproteins. Numerous studies have demonstrated the efficacy of high AIP values in predicting the risk of CVD (Elmugadam et al., 2022). However, studies examining the relationship between AIP and bone mineral density in postmenopausal women are quite limited, and the existing results are inconsistent (Hernández et al., 2021). In a similar manner, the triglyceride-glucose (TyG) index, calculated using the following formula: ln(triglycerides × fasting blood glucose/2), is considered a strong marker for insulin resistance. Insulin resistance has been demonstrated to contribute to both vascular endothelial dysfunction and bone turnover imbalance, thus creating a common pathophysiological basis for CVD and OP. Insulin has been shown to stimulate the proliferation of osteoblasts and collagen synthesis; however, insulin resistance has been shown to impair bone formation by reducing these effects. The TyG index has been demonstrated to be significantly associated with CVD and poor prognosis (Zhou et al., 2023). Recent cross-sectional studies have shown that a high TyG index is associated with low BMD and increased OP risk in postmenopausal women (Korpe et al., 2025). However, the underlying mechanisms of this relationship and the clinical value of the TyG index in OP screening have yet to be elucidated.

Chronic inflammation plays a significant role in the pathogenesis of OP. In this context, systemic inflammation indices derived from complete blood counts have emerged as rapid and cost-effective potential markers reflecting postmenopausal OP risk. Several studies have investigated markers such as the neutrophil-lymphocyte ratio (NLR), platelet-lymphocyte ratio (PLR), monocyte-lymphocyte ratio (MLR), and neutrophil-monocyte ratio (NMR) (Demir Cendek et al., 2024). For instance, a recent meta-analysis reported a significant association between elevated NLR and PLR values and postmenopausal OP (Liu et al., 2022). These markers have also been correlated with cardiovascular risk in postmenopausal women, supporting the inflammatory link between bone and vascular health (Qu et al., 2023).

Beyond simple inflammatory ratios, novel metabolic markers that combine immune cell counts with lipid profiles—specifically high-density lipoprotein cholesterol (HDL-C)—are gaining attention. Ratios such as lymphocyte/HDL-C, monocyte/HDL-C, and granulocyte/HDL-C have been associated with both postmenopausal OP and asymptomatic CVD (Carranza-Lira et al., 2020; Huang et al., 2022). Given the anti-inflammatory and antioxidant properties of HDL-C, these composite indices may offer a more comprehensive risk stratification by reflecting both immune dysregulation and an atherogenic lipid profile compared to traditional markers alone. Consequently, these HDL-associated markers represent a potential new approach for OP screening.

Despite these findings, comprehensive studies evaluating the collective diagnostic performance of all these indices in postmenopausal OP are quite limited. Although dual-energy X-ray absorptiometry (DXA) remains the gold standard for OP diagnosis, its limitations, such as high cost, radiation exposure, and limited accessibility, necessitate the development of alternative screening tools. While these biomarkers currently lack the capacity to replace BMD measurement for definitive OP diagnosis, they have been proposed as accessible and cost-effective candidate markers for risk stratification; however, their value as screening tools remains uncertain because the available evidence is inconsistent. Therefore, the present study sought to ascertain the relationship between inflammatory and metabolic indices (NLR, PLR, MLR, NMR, lymphocyte/HDL-C, monocyte/HDL-C, granulocyte/HDL-C, AIP, and TyG) and postmenopausal OP, and to evaluate their diagnostic discrimination for postmenopausal OP.

## Materials & Methods

**Figure 1:**
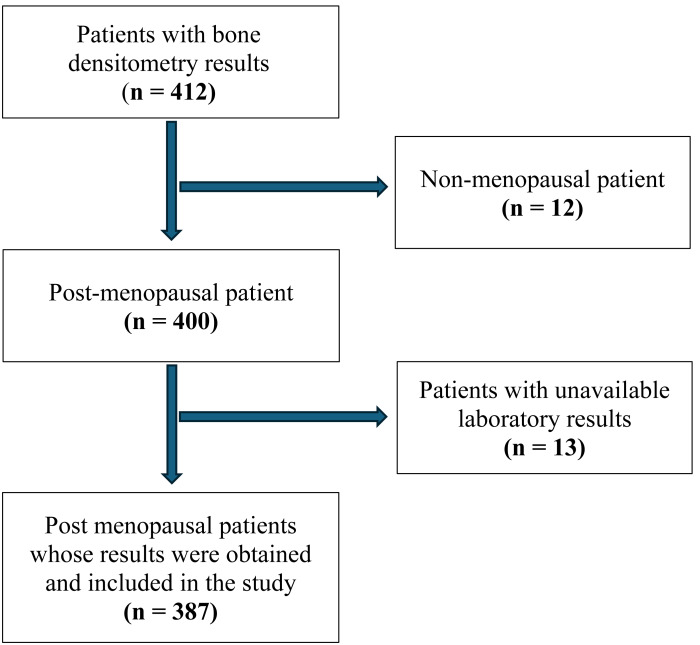
The flowchart of the experimental design.

For this study, bone densitometry results were collected from 412 patients who visited the gynaecology and menopause outpatient clinic of Bandırma Onyedi Eylül University Hospital between 2021 and 2025 and requested bone densitometry due to suspicion of OP. Upon analysing the outpatient clinic records, 12 patients were not in menopause and were excluded from the study. The haemogram and lipid profile results for 13 of the remaining 400 patients were unavailable, and these patients were also excluded. The remaining 387 postmenopausal patients were included in the study. The study selection process is illustrated in the flowchart in Fig. 1.

### Type (design) of the study

This retrospective diagnostic accuracy study was carried out through a file and archive review.

### Patient informed consent

Because this study was conducted as a retrospective file review, the requirement for informed consent was waived by the relevant ethics committee. Permission to use the data was obtained from the Education Planning Committee of Bandırma Onyedi Eylül University Hospital (approval number: E-44767171-799-288458238).

### Data collection method

Bone densitometry examinations of the patients were accessed through patient information system records. The bone densitometry results included vertebral and femur T-scores, as well as the patient’s age, menopausal status, body weight, and height data, which were collected during the scan and reported. BMI was calculated by dividing body weight by the square of height (Rakıcıoğlu, Samur & Başoğlu, 2017). The data collected were documented on the patient data form. Laboratory parameters including haemogram, high density lipoprotein (HDL), triglyceride (TG), and fasting blood sugar (FBS) were collected from tests performed on the same day as the bone densitometry examination. This simultaneous measurement approach ensured optimal temporal correlation between biomarker levels and BMD assessment. Femoral neck measurement was selected for group classification because isolated vertebral osteoporosis was not observed in the study cohort. The proximal ends of the femur are the most susceptible areas to osteoporotic fractures compared to the spine and other parts of the bone, and hip fracture is considered the most serious complication of OP, associated with high morbidity and mortality (Nuti et al., 2019; Osterhoff et al., 2016).

NLR, PLR, MLR, and NMR values are derived from neutrophil, lymphocyte, platelet, monocyte, and granulocyte counts in the complete blood count. AIP is calculated using TG and HDL-C values. The TyG index is determined using fasting blood glucose and TG levels. Ratios such as lymphocyte/HDL-C, monocyte/HDL-C, granulocyte/HDL-C, and HDL-C are calculated from respective cell counts and HDL-C levels. Systemic Immune Inflammation Index (SII), Systemic Inflammatory Response Index (SIRI), and Aggregate Index of Systemic Inflammation (AISI) were calculated using the formulas found in the literature (Liu et al., 2022; Caimi et al., 2024; Zhou et al., 2016; Li et al., 2024; Zhu & Yuan, 2025).

### Ethics committee approval

Ethical approval was obtained from Bandırma Onyedi Eylül University Health Sciences Non-Interventional Research Ethics Committee (Date: 23.05.2025, Decision No: 2025-108) Document No.: E-25691463-050.04-2500026609.

### Data analyses

The data were evaluated in the SPSS 23.0 statistical package programme. The conformity of continuous data to normal distribution was evaluated by the Kolmogrow Smirnow test. Median and interquartile range (IQR) values were presented for continuous data that did not conform to normal distribution. The Kruskal-Wallis test was used to compare the baseline characteristics, clinical characteristics, biochemical parameters, and immunological parameters of participants with normal bone density, those with osteopenia, and those with OP according to FN T-score. Using the bone densitometry results, a vertebral and/or FN T-score <−2.5 was considered OP, an FN T-score between -1 and −2.5 was considered osteopenia, and an FN T-score >−1 was considered normal. Dunn’s multiple comparison test was used for intergroup comparisons. The relationship between biochemical and immunological parameters and FN T-score was examined by Spearman correlation analysis, and the results were presented with scatter plots. Receiver operating characteristic (ROC) curves, area under the curve (AUC) and sensitivity, specificity, positive likelihood ratio (+LR), and negative likelihood ratio (-LR) were calculated to evaluate the diagnostic performance of biochemical and immunological parameters. AUC values of 0.9−1.0 were considered excellent, 0.8−0.89 good, 0.7−0.79 fair, 0.6−0.69 poor, and less than 0.6 were considered fail values (Dirican, 2001; Zou, O’Malley & Mauri, 2007; Hanley & McNeil, 1983). In determining the optimal cut-off points of biochemical and immunological parameters for the diagnosis of OP, the Youden index was calculated, and the highest index value was determined to be the cut-off point. The significance level of statistical tests was accepted as *p* < 0.05.

## Results

The distribution of some demographic and clinical patient characteristics between the normal, osteopenia, and OP groups according to bone mineral density is presented in Table 1. Age and menopause duration were significantly higher in the OP group compared to the normal and osteopenia groups (*p* < 0.01). Conversely, the median FN T-score was significantly lower in the OP group compared to the other two groups. The median value of height was significantly lower in the OP group (median: 1.58, IQR: 1.54−1.63) than in the normal (median: 1.62, IQR: 1.58−1.65) and osteopenia (median: 1.60, IQR: 1.57−1.65) groups (*p* < 0.01, Table 1).

**Table 1:** Distribution of some basic and clinical characteristics among normal, osteopenia and osteoporosis groups according to bone mineral density.

**Features**	**All groups** **(*n* = 387)**	**Normal** **(T-score ≥−1.0)** **(*n* = 94)**	**Osteopenia** **(−2.5<T-score <−1.0)** **(*n* = 219)**	**Osteoporosis** **(T-score ≤−2.5)** **(*n* = 74)**	***p*** ^*^
	**Median** **(IQR)**	**Median** **(IQR)**	**Median** **(IQR)**	**Median** **(IQR)**	
Age (Year)	54.00 (50.00–61.00)	51.00 (47.00–55.00)^a^	53.00 (50.00–60.00)^b^	61.00 (54.00–69.25)^c^	<0.001
Body weight (kg)	69.00 (62.00–75.00)	70.00 (61.00–78.25)	70.00 (63.00–75.00)	65.50 (60.00–71.25)	0.056
Height (m)	1.60 (1.57–1.65)	1.62 (1.58–1.65)^a^	1.60 (1.57–1.65)^a^	1.58 (1.54–1.63)^b^	<0.001
BMI (kg/m^2^)	26.44 (24.12–29.17)	26.28 (23.90–29.42)	26.45 (24.34–29.29)	27.12 (23.76–29.14)	0.948
Duration of menopause (Year)	6.00 (2.00–15.00)	4.00 (1.00–9.25)^a^	6.00 (2.00–14.00)^b^	15.00 (7.75–22.00)^c^	<0.001
FN T-score	−1.50 (−1.00–2.20)	−0.50 (–0.70 to −0.03)^a^	−1.60 (−2.00 to −1.30)^b^	−2.70 (−2.90 to −2.50)^c^	<0.001

**Notes.**

BMI Body Mass Index FN Femoral Neck IQR Interquartile Range

* Kruskal–Wallis test with Dunn’s multiple comparisons test. Superscripts a, b, and c indicate differences between groups on the same line. There is no statistically significant difference between groups with the same superscripts.

Table 2 shows the distribution of biochemical and immunological parameters among the normal, osteopenia, and OP groups according to bone mineral density. There was no statistically significant difference between the normal, osteopenia, and OP groups in terms of median value of NLR, PLR, MLR, NMR, AIP, TyG Index, lymphocyte/HDL-C, granulocyte/HDL-C, SII, SIRI, or AISI (*p* > 0.05). The median value of monocyte/HDL-C was significantly lower in the OP group (median: 0.007, IQR: 0.005−0.010) compared to the normal (median: 0.009, IQR: 0.007−0.011) and osteopenia (median: 0.009, IQR: 0.006−0.012) groups (*p* < 0.05, Table 2).

**Table 2:** Distribution of biochemical and immunological parameters among normal, osteopenia and osteoporosis groups according to bone mineral density.

**Parameters**	**All groups** **(*n* = 387)**	**Normal** **(T-score ≥−1.0)** **(*n* = 94)**	**Osteopenia** **(−2.5<T-score <−1.0)** **(*n* = 219)**	**Osteoporosis** **(T-score ≤−2.5)** **(*n* = 74)**	***p*** ^*^
	**Median** **(IQR)**	**Median** **(IQR)**	**Median** **(IQR)**	**Median** **(IQR)**	
NLR	1.59 (1.200–2.140)	1.630 (1.280–2.060)	1.590 (1.180–2.270)	1.530 (1.170–1.970)	0.547
PLR	113.470 (90.900–139.580)	109.820 (93.520–139.470)	116.230 (90.910–140.690)	109.260 (89.230–132.770)	0.485
MLR	0.210 (0.160–0.270)	0.200 (0.180–0.290)	0.210 (0.170–0.270)	0.200 (0.170–0.250)	0.126
NMR	7.650 (6.130–9.410)	7.690 (6.080–8.980)	7.510 (5.880–9.680)	7.950 (6.710–9.630)	0.639
AIP	2.020 (1.330–3.320)	2.030 (1.310–3.540)	2.140 (3.48–1.34)	1.770 (1.350–2.830)	0.222
TyG Index	8.720 (8.340–9.210)	8.630 (8.350–9.080)	8.780 (8.340–9.240)	8.670 (8.340–9.060)	0.072
Lymphocyte/HDL-C	0.038 (0.029–0.051)	0.039 (0.054–0.030)	0.039 (0.052–0.029)	0.037 (0.047–0.027)	0.662
Monocyte/HDL-C	0.008 (0.005–0.011)	0.009 (0.007–0.011)^a^	0.009 (0.006–0.012)^a^	0.007 (0.005–0.010)^b^	0.009
Granulocyte/HDL-C	0.066 (0.048–0.093)	0.068 (0.054–0.094)	0.068 (0.045–0.098)	0.062 (0.044–0.086)	0.321
SII	421.199 (306.881–593.857)	454.801 (5325.049–68.226)	422.702 (303.771–633.288)	404.872 (291.695–551.261)	0.719
SIRI	0.803 (0.534–1.173)	0.795 (0.571–1.190)	0.843 (0.525–1.245)	0.769 (0.512–0.999)	0.599
AISI	211.402 (133.289–337.913)	224.467 (142.230–336.370)	221.296 (132.179–359.584)	188.087 (131.186–292.709)	0.113

**Notes.**

NLR Neutrophil Lymphocyte Ratio PLR Platelet Lymphocyte Ratio MLR Monocyte Lymphocyte Ratio NMR Neutrophil Monocyte Ratio AIP Atherogenic Index of Plasma TyG Index Triglyceride Glucose Index Lymphocyte/HDL-C Lymphocyte High Density Lipoprotein Ratio Monocyte/HDL-C Monocyte High Density Lipoprotein Ratio Granulocyte/HDL-C Granulocyte High Density Lipoprotein Ratio SII Systemic Immune Inflammation Index SIRI Systemic Inflammatory Response Index AISI Aggregate Index of Systemic Inflammation IQR Interquartile Range

* Kruskal–Wallis test with Dunn’s multiple comparisons test. Superscripts a, b, and c indicate differences between groups on the same line. There is no statistically significant difference between groups with the same superscripts.

**Figure 2:**
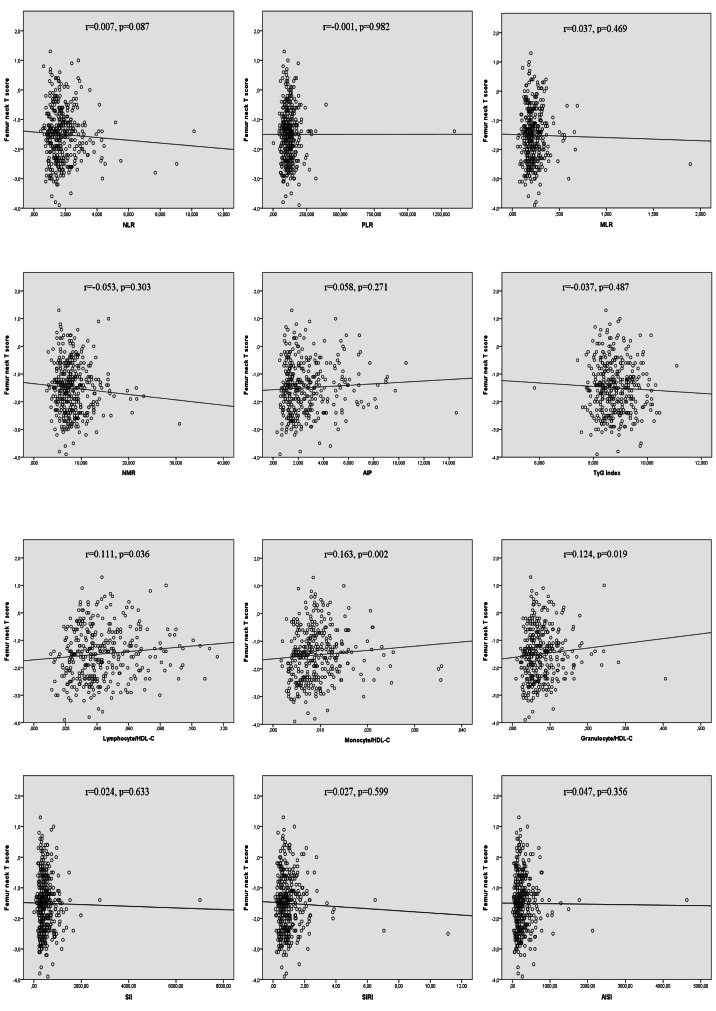
The relationship between biochemical and immunological parameters and femoral neck T score.

Figure 2 shows the relationship between biochemical and immunological parameters and FN T-score. In a Spearman correlation analysis, a statistically significant positive weak correlation was found between FN T-score and lymphocyte/HDL-C, monocyte/HDL-C, and granulocyte/HDL-C levels (*p* < 0.05). No correlation was found between FN T-score and NLR, PLR, MLR, NMR, AIP, TyG Index, SII, SIRI, or AISI (*p* > 0.05, Fig. 2).

**Figure 3:**
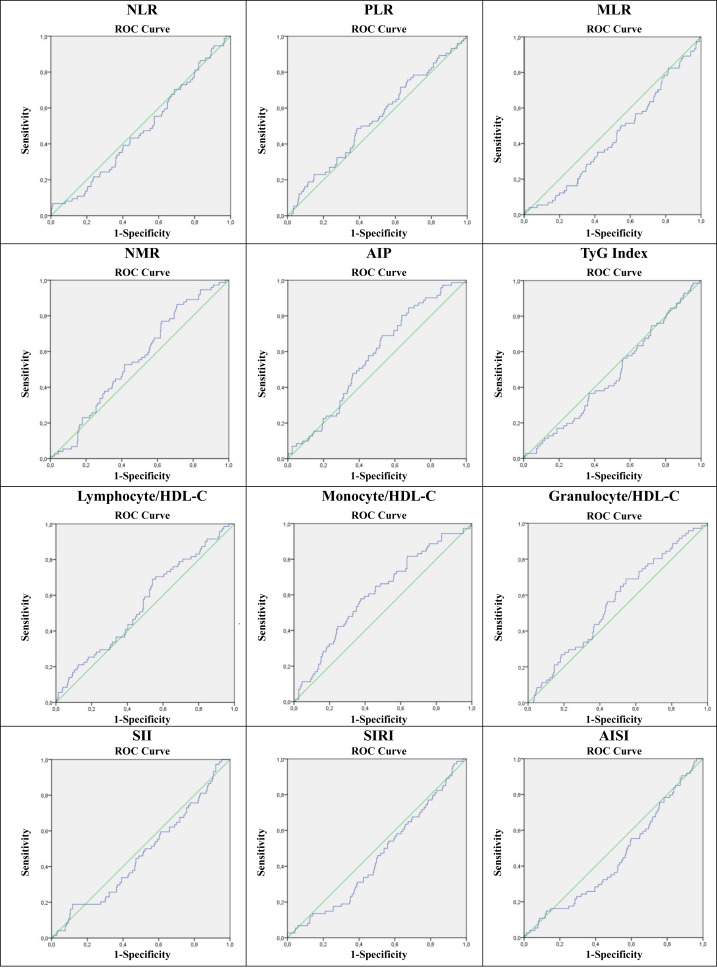
ROC curves of biochemical and immunological parameters in the prediction of osteoporosis.

Table 3 shows the diagnostic discrimination of biochemical and immunological parameters for distinguishing OP. The AUC calculated by ROC curves showed that monocyte/HDL-C (AUC: 0.608, *p* < 0.05) had a poor discriminatory ability and that NLR (AUC: 0.476), PLR (AUC: 0.537), MLR (AUC: 0.436), NMR (AUC: 0.554), AIP (AUC: 0.569), TyG Index (AUC: 0.473), lymphocyte/HDL-C (AUC: 0.552), granulocyte/HDL-C (AUC: 0.559), SII (AUC: 0.467), SIRI (AUC: 0.452), and AISI (AUC: 0.448) all other parameters also showed poor or non-significant discriminatory ability (Table 3). The ROC curves for these parameters are presented in Fig. 3.

**Table 3:** Diagnostic performance of biochemical and immunological parameters in the prediction of osteoporosis.

**Parameters**	**AUC** **(95%CI)**	**SE**	***p***	**Optimal cut-off value**	**Sensitivity** **(%)**	**Specificity** **(%)**	**(+) LR**	**(−) LR**	**Youden** **index**
NLR	0.476 (0.405–0.548)	0.036	0.529	4.328	0.068	0.990	7.049	0.941	0.058
PLR	0.537 (0.464–0.611)	0.037	0.318	106.213	0.486	0.613	1.258	0.837	0.100
MLR	0.436 (0.365–0.506)	0.036	0.087	0.591	0.027	0.990	2.819	0.982	0.017
NMR	0.554 (0.486–0.621)	0.034	0.151	6.176	0.865	0.291	1.219	0.464	0.156
AIP	0.569 (0.500–0.638)	0.035	0.070	3.060	0.845	0.323	1.248	0.479	0.168
TyG Index	0.473 (0.400–0.546)	0.037	0.481	7.860	0.986	0.045	1.032	0.312	0.031
Lymphocyte/HDL-C	0.552 (0.478–0.626)	0.038	0.176	0.041	0.690	0.458	1.274	0.676	0.148
Monocyte/HDL-C	0.608 (0.536–0.680)	0.037	**0.005**	0.008	0.577	0.625	1.539	0.676	0.202
Granulocyte/HDL-C	0.559 (0.487–0.631)	0.037	0.122	0.081	0.732	0.382	1.184	0.701	0.114
SII	0.467 (0.394–0.540)	0.037	0.380	765.658	0.189	0.882	1.600	0.919	0.071
SIRI	0.452 (0.382–0.523)	0.036	0.202	0.330	0.973	0.070	1.046	0.384	0.043
AISI	0.448 (0.376–0.519)	0.036	0.162	59.662	0.986	0.048	1.036	0.281	0.034

**Notes.**

NLR Neutrophil Lymphocyte Ratio PLR Platelet Lymphocyte Ratio MLR Monocyte Lymphocyte Ratio NMR Neutrophil Monocyte Ratio AIP Atherogenic Index of Plasma TyG Index Triglyceride Glucose Index Lymphocyte/HDL-C Lymphocyte High Density Lipoprotein Ratio Monocyte/HDL-C Monocyte High Density Lipoprotein Ratio Granulocyte/HDL-C Granulocyte High Density Lipoprotein Ratio SII Systemic Immune Inflammation Index SIRI Systemic Inflammatory Response Index AISI Aggregate Index of Systemic Inflammation AUC Area Under the Curve CI Confidence Interval SE Standard Error LR Likelihood Ratio

## Discussion

The prevalence of OP is rising with the ageing global population, leading to increased morbidity, mortality, and healthcare costs (Kanis et al., 1994; Johnell & Kanis, 2006). Emerging evidence suggests that chronic low-grade inflammation and lipid metabolism disorders play crucial roles in postmenopausal bone loss. Various inflammatory and metabolic biomarkers, including traditional ratios and composite indices such as the Systemic Immune Inflammation Index (SII), Systemic Inflammatory Response Index (SIRI), and Aggregate Index of Systemic Inflammation (AISI), have been proposed for risk assessment (Demir Cendek et al., 2024; Yilmaz et al., 2014; Zhang et al., 2023). However, studies examining these markers in relation to bone mineral density have yielded inconsistent results.

In this study, the relationship between bone mineral density and several biochemical and immunological markers of inflammation were thoroughly examined in postmenopausal women. The results of this study offer current and comprehensive data on how parameters such as NLR, PLR, MLR, NMR, AIP, TyG, lymphocyte/HDL-C, monocyte/HDL-C, granulocyte/HDL-C, SII, SIRI, and AISI relate to bone mineral density. Since all analyses in our study were unadjusted, the observed associations should be interpreted cautiously, as they may be influenced by baseline differences between the groups, particularly age and menopause duration.

### Comparison and interpretation of findings with the literature

The results of this study indicated that there was no significant difference between the groups regarding parameters such as NLR, PLR, MLR, NMR, AIP, TyG, lymphocyte/HDL-C, granulocyte/HDL-C, SII, SIRI, and AISI. However, the monocyte/HDL-C ratio was significantly lower in the OP group compared to the other groups and demonstrated a weak positive correlation with BMD. The ROC analysis revealed that the monocyte/HDL-C ratio had a statistically significant but limited discriminatory ability to distinguish women with OP from other groups.

The relationship between NLR, PLR, and MLR, which are traditional markers of inflammation, and bone mineral density has been widely discussed in the literature. Some studies have reported that these ratios are inversely correlated with BMD, but the evidence remains neither statistically strong nor consistent (Yilmaz et al., 2014). For example, multicentre studies such as Demir Cendek et al. (2024) and Al Salmani et al. (2022) have shown that NLR, PLR, and MLR have low sensitivity in diagnosing OP, and their use alone is limited. Yolaçan & Guler (2023) stated that these inflammatory markers may be significant in some subgroups, but their effect on the general population is limited.

Composite inflammation indices (SII, SIRI, AISI) demonstrate more consistent and stronger relationships in the literature. Many studies report that SII is significantly and inversely associated with BMD, with higher SII values linked to lower BMD. For example, one study found the AUC value of SII to be 0.686 (Bolkan Günaydin & Ay, 2022), and and another one was reported that a doubled SII value was associated with a 1.4−1.7% decrease in BMD (Zhang et al., 2023). Additionally, new composite indices such as Pan-Immune-Inflammation Value (PIV) have shown OR values between 5.99 and 7.63 in the prediction of OP (Başaran & Tutan, 2024).

Regarding lipid-related parameters, the TyG index, monocyte/HDL-C (MHR), and apolipoprotein A1 (MAR) were found to be inversely related to BMD. The AUC values for the TyG index ranged from 0.767 to 0.818, while the AUC of MAR was found to be 0.843. The odds ratios for these parameters varied between 1.88 and 2.30 (Korpe et al., 2025; Huang et al., 2022). However, no single marker alone possesses high sensitivity and specificity.

### Clinical significance of biochemical and immunological parameters

In clinical practice, early diagnosis and risk prediction of postmenopausal OP are crucial for disease prevention and reducing disease complications. Consequently, there is a significant need for accessible, low-cost, and objective biomarkers for postmenopausal OP. Although complete blood count-based inflammatory markers (such as NLR, PLR, MLR) and recently defined composite indices (SII, SIRI, AISI) appear promising, this study and similar research indicate that these parameters alone do not demonstrate sufficient diagnostic performance for routine clinical use in postmenopausal OP.

Although traditional markers of inflammation reflect the systemic inflammatory state, their effects on bone metabolism are indirect and can be influenced by many factors. For instance, conditions such as infections, chronic diseases, drug use, and even psychosocial stress can modify these inflammatory markers. Moreover, the impact of inflammation on bone is typically a long-lasting and low-grade process rather than an immediate one. Therefore, it is likely that inflammatory markers measured at a single time point would not show a strong association with bone mineral density.

In recent years, lipid-related parameters have begun to play a significant role in bone health in patients with metabolic syndrome, diabetes, and cardiovascular diseases. Notably, the MHR has gained prominence because it indicates both inflammation and lipid profile. However, the diagnostic value of this ratio is limited, and it does not offer high accuracy on its own.

It is noteworthy that in our study, the monocyte/HDL-C ratio was found to be lower in the OP group, contrary to the general expectation in the literature of an association with increased inflammation (Li et al., 2024). This paradoxical finding may be explained by the significantly higher mean age of the OP group (*p* < 0.01) and the consequent higher probability of statin or anti-inflammatory medication use due to comorbidities. As is known, statins can artificially lower monocyte/HDL-C ratios by suppressing monocyte counts and increasing HDL levels (Abut4ht=&CÇ Aktürk, 2021). The inability to definitively exclude medication history (statins, bisphosphonates, or HRT) due to the retrospective nature of our study represents a limitation that may have influenced these inflammatory markers (Sanllorente et al., 2021).

Additionally, the OP group exhibited significantly lower height compared to the normal group (Table 1). While low constitutional stature is a known risk factor for osteoporosis, this height deficit may also reflect height loss secondary to osteoporotic vertebral compression fractures, which are common in this older demographic. Therefore, the anthropometric differences observed should be interpreted as both a potential cause and a consequence of the disease.

Compound inflammation indices (SII, SIRI, AISI) aim to be a more holistic indicator of systemic inflammation by evaluating multiple cellular components together. However, the diagnostic performance of these indices is limited. This indicates that bone tissue is affected by many factors other than inflammation, such as hormonal, genetic, nutritional, and lifestyle factors, and it is difficult to predict the risk of OP with a single biomarker.

### Inconsistencies in the literature and possible causes

While previous studies have reported significant associations between inflammatory markers and osteoporosis, our findings present a notable contrast. For instance, Yilmaz et al. (2014) and Zhang et al. (2023) suggested that markers like NLR and SII possessed strong diagnostic value. However, our study did not demonstrate similarly high discriminatory ability in a general older population. This discrepancy likely stems from the high sensitivity of these biomarkers to demographic heterogeneity. Unlike studies that may have excluded patients with common metabolic comorbidities, our cohort represents a ‘real-world’ clinical sample with a higher median age and associated medication usage. Consequently, our negative results serve as a critical caution: these biomarkers are not universally reliable across all postmenopausal subgroups and their diagnostic utility appears strictly limited to specific populations, rather than serving as broad screening tools.

### Future research directions

Future studies in this field should address current limitations and advance our understanding through larger and more homogeneous sample groups and multicentre and prospective designs with diverse age, ethnicity, and comorbidity profiles to enhance generalisability. Longitudinal follow-up approaches are essential, since the effect of inflammatory markers on BMD is spread over time, making long-term and repeated measurements more likely to reveal meaningful relationships.

The development of multifactorial risk models that integrate clinical, laboratory, and imaging findings, supported by new technologies such as artificial intelligence and machine learning, should be prioritised. Subgroup analyses should evaluate the discriminatory ability of inflammation and lipid markers individually, particularly in individuals with additional risk factors such as diabetes, obesity, and cardiovascular disease. Dynamic monitoring of biomarkers by tracking changes over time may be more beneficial than relying on a single measurement, especially in predicting the rate of bone loss.

Through these comprehensive methodological improvements and technological advances, future research may ultimately clarify the clinical utility of biochemical and immunological markers in postmenopausal OP management and contribute to the development of more precise, personalised diagnostic and treatment strategies.

### Study limitations

The primary limitation of this study is its retrospective design and the fact that all analyses were unadjusted. Accordingly, the observed associations should not be interpreted as independent effects. In particular, age and duration of menopause were significantly higher in the osteoporosis group and may have confounded the relationships between the evaluated biomarkers and bone mineral density. In addition, we were unable to access detailed medication histories, specifically regarding the use of statins, hormone replacement therapy, and bisphosphonates. Since these medications may directly influence both lipid-related and inflammatory parameters as well as bone metabolism, the lack of these data further limits interpretation. Lifestyle factors such as dietary calcium/vitamin D intake, physical activity, and smoking status could also not be accounted for. Therefore, our findings should be interpreted as preliminary, unadjusted associations that require validation in prospective studies with appropriate multivariable adjustment.

## Conclusions

Although the monocyte/HDL-C ratio demonstrated statistical significance among the evaluated parameters, its diagnostic discrimination remained limited (AUC: 0.608). Given that this performance falls below the threshold required for clinical utility, our findings suggest that these biomarkers are currently insufficient to be recommended as standalone screening tools for OP.

In conclusion, although inflammation and lipid-related biomarkers are promising for the diagnosis and risk prediction of postmenopausal OP, they are not sufficient on their own based on current evidence. These easily accessible and cost-effective parameters should be assessed alongside traditional risk factors in a comprehensive and personalised approach, serving as supplementary tools rather than standalone diagnostic markers for identifying individuals at risk of OP. Future prospective studies with larger sample sizes may help clarify the optimal role of these biomarkers in clinical practice.

##  Supplemental Information

10.7717/peerj.21311/supp-1 Supplemental Information 1 Raw data

## Additional Information and Declarations

### Data Availability

The following information was supplied regarding data availability: The raw measurements are available in the Supplemental File.
